# Host–Guest Extraction of Heavy Metal Ions with* p*-*t*-Butylcalix[8]arene from Ammonia or Amine Solutions

**DOI:** 10.1155/2018/4015878

**Published:** 2018-07-11

**Authors:** Md. Hasan Zahir, Shakhawat Chowdhury, Md. Abdul Aziz, Mohammad Mizanur Rahman

**Affiliations:** ^1^Center of Research Excellence in Renewable Energy, Research Institute, King Fahd University of Petroleum and Minerals, Dhahran 31261, Saudi Arabia; ^2^Department of Civil and Environmental Engineering, Water Research Group, King Fahd University of Petroleum and Minerals, Dhahran 31261, Saudi Arabia; ^3^Center of Research Excellence in Nanotechnology, King Fahd University of Petroleum and Minerals, Dhahran 31261, Saudi Arabia; ^4^Center of Research Excellence in Corrosion, King Fahd University of Petroleum and Minerals, Dhahran 31261, Saudi Arabia

## Abstract

The capacities of the* p*-*t*-butylcalix[8]arene (abbreviated as H_8_L) host to extract toxic divalent heavy metal ions and silver from aqueous solution phases containing ammonia or ethylene diamine to an organic phase (nitrobenzene, dichloromethane, or chloroform) were carried out. When the metal ions were extracted from an aqueous ammonia solution, the metal ion selectivity for extraction was found to decrease in the order Cd^2+^> Ni^2+^> Cu^2+^> Ag^+^> Co^2+^> Zn^2+^. When the aqueous phase contained ethylene diamine, excellent extraction efficiencies of 97% and 90% were observed for the heavy metal ions Cu^2+^ and Cd^2+^, respectively. Under the same conditions the extraction of octahedral type metal ions, namely, Co^2+^ and Ni^2+^, was suppressed. The extraction of transition metal cations by H_8_L in ammonia and/or amine was found to be pH dependent. Detailed analysis of extraction behavior was investigated by slope analysis, the continuous variation method, and by loading tests.

## 1. Introduction

Host–guest chemistry has attracted great attention in the field of separation and/or extraction of alkaline, rare earth, and divalent heavy metal ions with calixarenes and their ester derivatives. Calixarenes have unusual capabilities to identify and distinguish between different ions and molecules, which makes them appropriate to use as specific receptors [1–3]. Calixarenes are macrocyclic phenolic oligomers with phenolic hydroxyl groups, which are able to coordinate the metal ions very tightly. As a result, the aromatic phenolic rings can form a cavity to integrate the guest metal ion. Recently, Yusof et al. reported that the heavy metal ions could be included into the host calix[4]resorcinarenes cavity in water-chloroform extraction systems [4, 5]. Calix[6]arenes can be also modified with a carboxylic acid as host in the* host*–*guest* extraction of immunoglobulin G (IgG) [6]. In fact, a precise affinity can be created for specific ions and/or molecules by modification the hydroxyl functional group and/or by creating a new cavity size [7]. In calixarenes, the cavity size, position and type of donor groups, and molecular flexibility lead to their high potential for the complexation and extraction of metal ions. Ludwig et al. reported the impact of calixarenes in analytical chemistry and chemical separation technology in a review article [8].

The modification of* p*-*t*-butylcalix[*n*]arenes (n = 4, 6, 8) has been extensively investigated, facilitating the synthesis of a variety of host compounds with varying shapes and sizes that have been shown to be valuable in ion or molecular recognition studies [9, 10]. In particular,* p*-*t*-butylcalix[8]arene (abbreviated as H_8_L) has shown interesting complexing properties towards C_60_-fullerene, cesium, or strontium cations. This means that new synthetic routes with different functionalized derivatives of H_8_L could be more versatile [11].

H_8_L can form host–guest complexes through hydrophobic and *π*–*π* interactions within the cavities of *π*-donors composed of benzene rings, polycyclic aromatic hydrocarbons, anthraquinones, phenol regioisomers, and fullerene *π*-systems, due to their considerable electron affinity [12]. From a coordination standpoint, the reaction of H_8_L with metal ions is more complex than that of H_6_L; however, already few calix[8]crowns have been reported in the open literature [13, 14]. Derivatives of calix[n]arenes could be formed by inserting binding groups such as amines and/or alcohols into the upper and lower rim positions of the calixarene moiety [15]. It has been reported that proton transfer from an OH-group of the parent calixarene to an amine might occur during the reaction period, eventually resulting in a new compound from the association and inclusion of the amine into the cavity of the calixarene [16]. Finally, the abovementioned studies have established that calixarenes can effectively react with most metal ions and the new organometallic compounds can be obtained in good yields [8–10, 17].

H_8_L has been utilized in the separation of metallic cations [18, 19], the extraction of methyl esters of some amino-acids [20], uranium (VI) preconcentration [21], lanthanide complexes [22], and molecular recognition of 1,5-diaminoantraquinone [23]. Erdemir et al. investigated the extraction abilities of carboxylic acid and methyl ester derivatives of* p*-*t*-butylcalix[n]arenes (n = 6, 8) for carcinogenic aromatic amines [24]. Other calixarenes and their derivatives have been utilized in the fields of complexation, separation, electroanalysis, spectroscopy, and chemometrics [25].

A larger ligand, i.e., H_8_L, can act as a ditopic receptor for lanthanide and transition metal ions [26] and hence in principle may bind to a single metal ion in various ways. It is important to mention that solvent extraction of transition metal ions, particularly toxic metal ions, using H_8_L alone has scarcely been reported in the open literature. Makrlik et al. studied the liquid–liquid extraction of Eu^3+^ trifluoromethanesulfonate into nitrobenzene in the presence of H_6_L and H_8_L [27]. Sansone et al. reported the separation of An^3+^/Ln^3+^ from radioactive waste using CMPO (carbamoylmethyl-phosphine oxide) substituted H_6_L and H_8_L [28]. Gutsche et al. synthesized aminocalixarenes complexes of Ni^2+^, Cu^2+^, Pd^2+^, Co^2+^, and Fe^2+^ and determined their spectral and chemical characteristics [10]. Their findings indicated that metal-aminocalixarenes are more flexible than had previously been thought. It has also been reported that H_8_L can be combined into a polymeric medium to produce a material that shows a high sorption ability towards transition metal ions (Cu^2+^, Fe^2+^, Zn^2+^, Ni^2+^, Co^2+^, Cd^2+^, and Pb^2+^) in aqueous solution [29]. The authors also performed research on the extraction behavior of transition metal ions with H_4_L and H_6_L [30, 31].

An effective extractant with high selectivity for metal ions is in high demand for analytical applications, recycling of resources, and waste treatment purposes. Heavy metals such as lead (Pb), copper (Cu), and nickel (Ni) are harmful to humans. Obviously, the harmful impact of some ions, for example, Cd^2+^ and As^2+^, is of great concern in such research. Cd^2+^ is one of the most toxic elements for humans. At high concentrations, it causes various debilitating conditions such as painful bone disease, bone marrow disorders, kidney problems, and “*Itaiitai” *or* “ouch-ouch” *disease [32]. However, calixarene derivatives may be useful binders for these cations.

In this study, H_8_L has been investigated as host extractant for divalent heavy metal ions and silver from ammonia and amine solutions into various types of organic solvents. We also synthesized H_8_L ethyl ester derivatives and the extraction behavior of transition metal cations from aqueous solution was also investigated.

## 2. Experimental Section

### 2.1. Materials

H_8_L was purchased from the Sigma Aldrich Chemical Company, USA. All transition metal nitrate solutions were prepared according to the method in [30, 31]. Other reagents such as chloroform, dichloromethane and nitrobenzene, and ethanol were purchased from Carlo Erba Reagents, France. Ammonia, ethylenediamine and trimethylene diamine, ethyl bromoacetate, NaH, and THF/DMF were bought from Acros Organic, Belgium. The water was deionized. All the remaining reagents were as pure as is commercially available. Stock solutions were standardized by potentiometric and EDTA titrations. Other metal salts were guaranteed to be reagent grade.

### 2.2. Extraction Procedure

The hosts H_8_L and H_8_L-ester were prepared by dissolving the appropriate amount in various organic solvents followed by the dilution, typically to 1 × 10^−4^ M working solution; aqueous solutions of metal solutions were made from analytical purity nitrates of Cd^2+^, Ni^2+^, Cu^2+^, Ag^2+^, Co^2+^, and Zn^2+^. Extraction experiments were typically performed by equilibrating 8 mL of a 5 × 10^−5^ M solution of the metal ions, 1 ml succinic acid (0.01M), and 1 mL of a buffer solution with 10 mL of a 5 × 10^−4^ M solution of the H_8_L in 1,2-dichloroethane. The mixture was placed in a stoppered 50 mL glass tube at a volume ratio of 10:10 mL (organic phase to aqueous phase). The pH was adjusted by three types of buffer solution: CH_3_COOH-CH_3_COONa (acidic region), H_3_BO_3_-NaOH (neutral), and NH_3_-NH_4_Cl (alkali). In the case of H_8_L-ester extractant, picric acid [2.5 × 10^−5^ M] was used as the counter anion. The extraction equilibrium was attained within 40 min of shaking with nitrobenzene; the extraction into chloroform reached equilibrium within 20 h of shaking. Therefore, the shaking time was fixed at 2 h for nitrobenzene and at 20 h for chloroform. The extractability was not affected by further shaking, indicating that the equilibrium was attained within 12 h. All the experiments were performed in presence of succinic acid to avoid emulsification during the extraction process. The distribution experiments were performed at room temperature.

Before shaking, the samples were left standing in the water bath at 25°C for 15 min to ensure that the extraction solutions were maintained at the same temperature. Then, a shaker at 200 stroke min^−1^ at 25.0 ± 0.1°C mixed the two phases; a shaking time of 12 h was sufficient to reach the extraction equilibrium. After shaking, the two phases were centrifuged at 2000 rpm. for 10 min, which was sufficient for complete separation. Before the measurement, the pH of the aqueous and organic phases was adjusted to 2.5 using 5 M HNO_3_ and 3 M LiOH. The amount of extracted metal ions was calculated from the difference between the metal concentrations in the aqueous phase before and after the equilibration. The concentration of the metal ion in the organic phase was determined by the back-extraction method; 5 cm^3^ of the organic phase was transferred into another glass-stoppered tube and shaken with 4 M hydrochloric acid. After phase separation, the equilibrium concentrations of metal cations in the aqueous phase were measured by an inductively coupled plasma atomic emission spectrometer (Seiko model SPS 1200AR). The equilibrium pH in the aqueous solutions was measured by a pH meter (Beckmann model ø45).

The extractability (Ex%) was determined from the decrease in the metal concentration in the aqueous phase:

(1) $$\begin{array}{c} Ex\%=\left\{\;\frac{{\left.\left(Metal\right)\right]}_{blank}–{\left[Metal\right]}_{water\left.\;\right)}}{{\left.\left(Metal\right)\right]}_{blank}}\;\right\}\times 100, \end{array}$$

where [Metal]_blank_ and [Metal]_water_ denote the metal concentrations in the aqueous phase after extraction with nitrobenzene and with the nitrobenzene solution containing extractants, respectively, and [Metal]_or_ denotes the metal concentration extracted into the organic phase.

### 2.3. Analysis

Morphology of the product particles was examined using scanning electron microscopy (SEM, JEOL JSM6330F). Fourier transform infrared (FT-IR) spectra were collected on a Bruker FT-IR spectrometer by using the KBr pellet technique. ^1^H-NMR data were recorded on a JEOL JNM-GX 61D FT-NMR spectrometer operating at 400 MHz in CDCl_3_, using TMS as internal standard.

## 3. Results and Discussion

### 3.1. Effect of H_8_L Host Concentration

Experiments were performed using H_8_L concentrations of 1 × 10^−3^ - 5 ×10^−4^ M and a metal ion concentration of 5 × 10^−5^ M; all other conditions were kept the same. We found that the extraction percentage increased with increasing H_8_L concentration. The best extraction was achieved when 5 × 10^−4^ M H_8_L was used. As H_8_L was soluble in nitrobenzene up to 4 × 10^−2^ M and up to 1 × 10^−2^ M in 1,2-dichloroethane at room temperature, the saturated solution was used as the stock solution.

### 3.2. Role of Extractant

Three organic solvents (nitrobenzene, dichloromethane, and chloroform) were also tested as inert diluents at a fixed pH for solutions containing an equal amount of metal ions and H_8_L. The phase volume ratio was maintained at 1:1 to avoid emulsion formation; this was found to be the most effective ratio. This means that the tendency for association is, in general, greater when the solvent–solute interactions are weaker; however, chloroform is the least effective. The exact cause of this type of behavior is not known. It was observed that the extraction percentage increased with the diluent type in the order of chloroform > dichloromethane > nitrobenzene.

### 3.3. Choice of Stripping Agent

After extraction of Cd^2+^ with H_8_L, the metal ions were stripped with 7 mL of various concentrations of mineral acid reagents, specifically 4 M HCl, 0.1–5 M HNO_3_, or 2 M H_2_SO_4_; lower concentrations of nitric acid (< 4.5 M) were not suitable as Cd^2+^ forms a stable complex. Finally, it was observed that 4 M HCl was suitable as a stripping agent.

### 3.4. Effect of Succinic Acid

Very small amounts of succinic was added to the reaction media to inhibit emulsification particularly in the case of Cd^2+^, Cu^2+^, and Cr^3+^ transition metal cations. By keeping all other parameters the same, experiments were performed using different concentrations of succinic acid. The best extraction was achieved when 0.01M succinic was used. It is noteworthy to mention that upon addition of the succinic acid into the highly basic buffer solution, the extraction percentage was stabilized probably due to the pH control.

### 3.5. Nature of the Extracted Species

**Figure 1:**
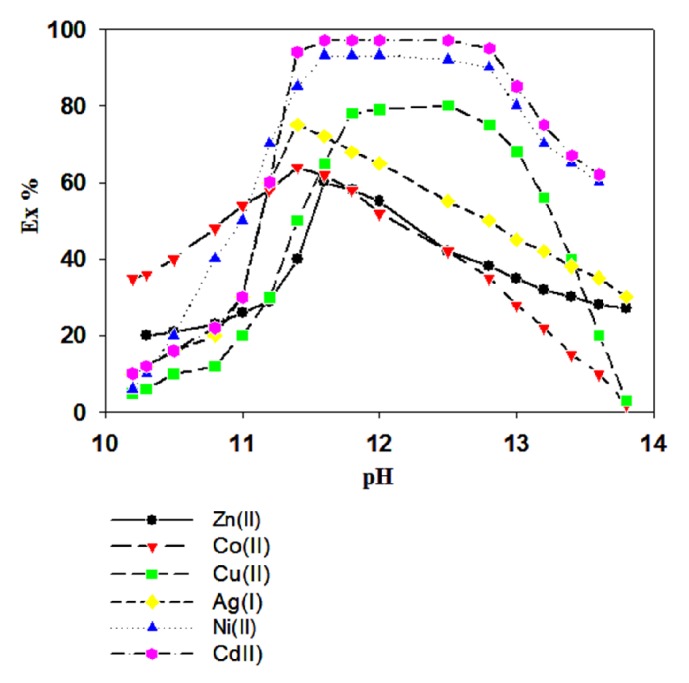
Extractability of all tested transition metal ions as a function of pH. Organic phase:* H*_*8*_*L* = 5 × 10^−4^ M; aqueous phase: = (●) 5 × 10^−5^ M; succinic acid = 0.01 M; and buffer solution: 0.01 M; MES-NaOH (pH 5.0–7.0) and 0.1 M Tris-HClO_4_ (pH 7.0–9.0). [Cd^2+^] = (■)1 × 10^−4^ M, 0.2 M NaClO_4_. O/A = 1; T = 25°C. O:A represents the ratio between organic (O) and aqueous (A) volumes in the experiments.

At first, we studied the effect of pH on the extraction of several transition metal ions with H_8_L in nitrobenzene. Specifically, the solvent extraction percentages of Cd^2+^, Ni^2+^, Cu^2+^, Ag^+^, Co^2+^, Zn^2+^, Cr^3+^, and Mn^2+^ transition metal cations were examined.  Figure 1 shows that the percentage of metal ions extracted increases as the pH increases from 10.0 to 13.0. In the acidic or neutral pH region, metal ions were not extracted with H_8_L, whereas at pH 11.3 more than 60% of metal ions were extracted with H_8_L. The maximum extraction percentage was observed in the pH range of the 11.5 to 13.00 for all tested samples. The extractability order is Cd^2+^ > Ni^2+^ > Cu^2+^ > Ag^+^ > Co^2+^ > Zn^2+^. H_8_L has phenolic hydroxyl groups which deprotonate at pH > ca.10 and deprotonated H_8_L can extract the metal ions. Among the cations tested, almost the same percentages of Cd^2+^ and Ni^2+^ ions were extracted under the experimental conditions. By contrast, no amounts of Cr^3+^ and Mn^2+^ were extracted, possibly due to the formation of a precipitate with ammonia solution. The results indicate that H_8_L has a good affinity for complexation with transition metal ions. The study of pH effect indicates that the extraction mechanism depends on a proton exchange mechanism together with hydrogen bonding. Petit et al. also reported a dinuclear cobalt(II) complex of calix[8]arenes compound, prepared by solvothermal reaction of cobalt(II) acetate with* p*-*t*-butylcalix[8]arene and trimethylamine; the compound was formed by hydrogen bond bridging [33].

**Figure 2:**
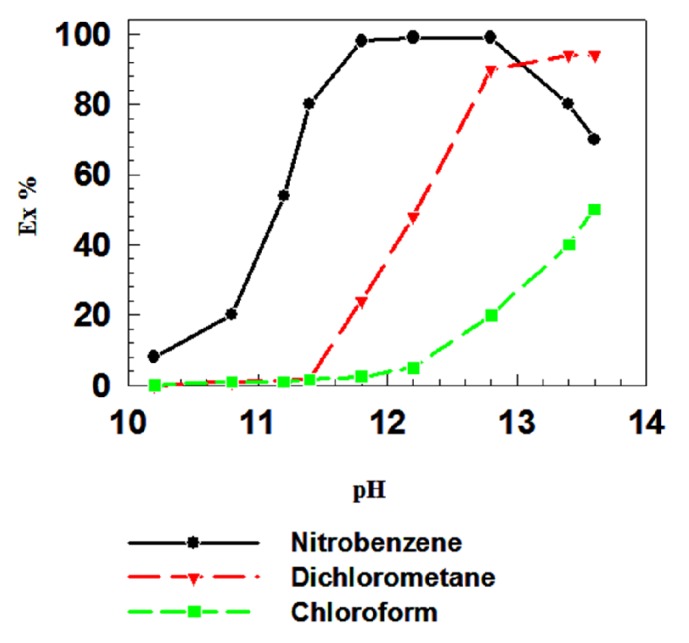
Effect of the organic solvent (nitrobenzene, dichloromethane, or chloroform) on the extraction percentage of Cd^2+^ with H_8_L.* H*_*8*_*L* = 5 × 10^−4^ M; aqueous phase [metal ion]: = (●) 5 × 10^−5^ M. O/A = 1; succinic acid = 0.01 M; and buffer solution, T = 25°C.

**Figure 3:**
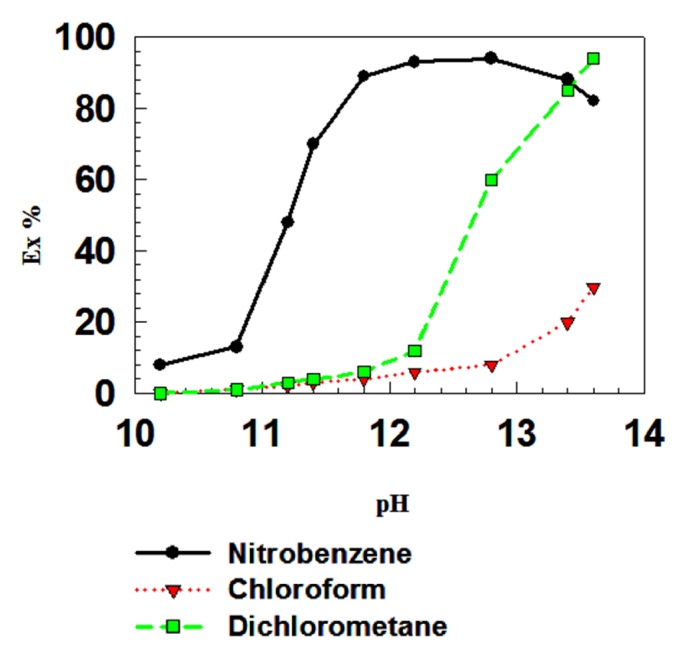
Effect of the organic solvent (nitrobenzene, dichloromethane, or chloroform) on the extraction percentage of Ni^2+^ with H_8_L.* p*-calix[8] = 5 × 10^−4^ M; aqueous phase [metal ion]: = (●) 5 × 10^−5^ M. succinic acid = 0.01 M and buffer solution, O/A = 1; T = 25°C.

The effects of three organic solvents, nitrobenzene, dichloromethane, and chloroform, were examined for both Cd^2+^ (Figure 2) and Ni^2+^ (Figure 3) in the pH range of 10-13.5. Nitrobenzene was very effective as an extractant for both Cd^2+^ and Ni^2+^ metal ions, extracting 96% of these metal ions in the pH range of 11.50-12.70. Dichloromethane was effective in the pH range 11.4-13 for Cd^2+^, whereas the effective pH range for Ni^2+^ was 12–13. Chloroform was less effective as an extractant at all pH values tested, showing only 50% extraction of Cd^2+^ and Ni^2+^ combined and similar extraction percentages for Cd^2+^ and Ni^2+^ individually. It has been reported that the H_6_L complex could be obtained as an adduct with chloroform (1 M); however, chloroform could not be removed from the adduct after calcination at 130°C for 3 days under reduced pressure. This indicates that the chloroform molecule might be encapsulated within the cavity of the calixarene and particularly for p-t-butyl calix[6]arene [H_6_L] molecule, which may account for the low extraction percentage obtained in the chloroform solution [25]. Three types of organic solvents were tested and the nitrobenzene is found to be the most efficient. Two other solvents, dichloromethane and chloroform, are least effective. The exact cause of this type of behavior is not known. Masuda et al. observed that the relative descending order of extraction with other solvents is not the same with H_6_L and the same order does not necessarily accord the order of their dielectric constants [31]. Actually the extraction percentages of Ce(III) with H_6_L were 95%, 27%, and 18% at pH 11.85 in the case of organic solvents nitrobenzene, dichloromethane, and chloroform. The dielectric contestants of nitrobenzene, dichloromethane, and chloroform are 34.82, 7.77, and 4.80, respectively. Therefore, it can be assumed that the dielectric contestant of the medium has some contribution in the extraction process. However, the main factor determining the extraction efficiency in the extraction process must be taken into account and a better term correlating the relative extraction order is solubility parameter. Moreover, Thuéry et al. reported that the reaction at room temperature between H_**8**_L and an excess of trimethylamine in chloroform provided a solid that could be recrystallized in methanol to yield dark red crystals of new compound suitable for X-ray crystallography. Therefore, we can conclude that the chloroform solvents have some affinity and/or suitable for complex formation with H_**8**_L ligand [34].

### 3.6. Slope Analysis

A traditional and effective means of obtaining both stoichiometric and equilibrium constant information about extraction processes, slope analysis, are based on an examination of the logarithmic variation of the distribution ratio, D, with relevant experimental variables. The log-log plots of the extraction in the form of D vs. a concentration variable indicate the stoichiometry of the formation of the extractable complex and thus lead to the derivation of a suitable equilibrium expression and then to the calculation of equilibrium constants.

**Figure 4:**
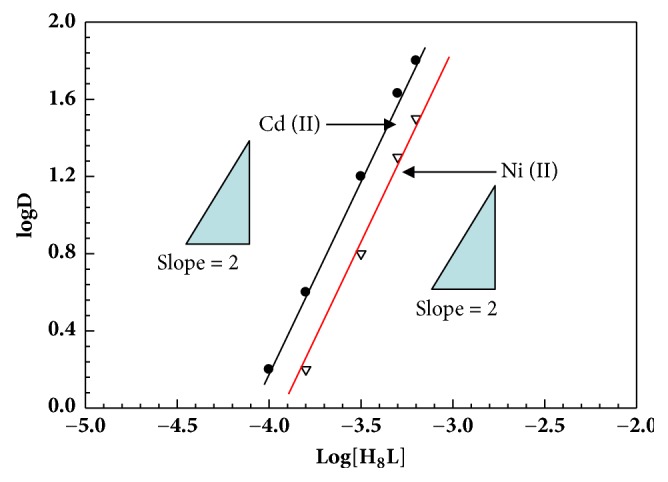
Plot of logD vs. log[H_n_L] for Cd^2+^ and Ni^3+^ under the same reaction condition of Figure 3.

**Figure 5:**
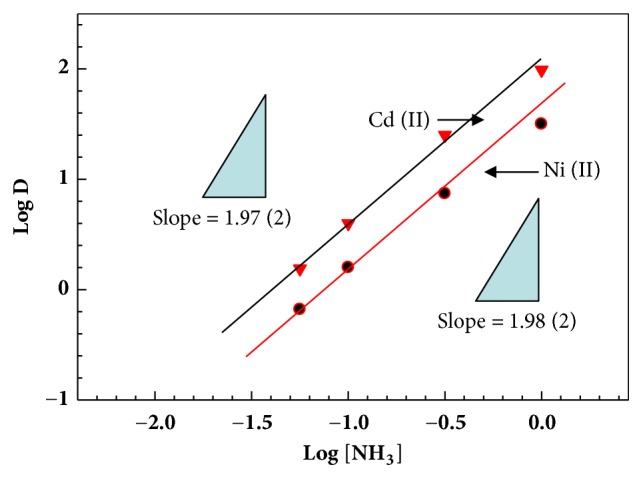
Plot of logD vs. log[NH_3_] for Cd^2+^ and Ni^3+^ with H_8_L under the same reaction condition of Figure 3.

Since the extraction reagents exhibited high selectivity for Cd^2+^ and Ni^2+^, detailed extraction behavior for Cd^2+^ and Ni^2+^ was investigated by slope analysis, the continuous variation method, and by loading tests. The extraction mechanism was studied by evaluating the composition of the extracted Cd^2+^ and Ni^2+^ species.  Figure 4 shows the effect of the initial concentration of H_8_L in the organic phase on the extraction of Cd^2+^ and Ni^2+^ from an aqueous ammonia solution. With increasing initial concentration of H_8_L, straight lines with a slope of 2 were obtained in the logD vs. log[H_8_L] plots for Cd^2+^ and Ni^2+^, indicating that the binding ratio of H_8_L with Cd^2+^ and Ni^2+^ is 2:1.  Figure 5 shows the effect of the initial concentration of ammonia in the aqueous phase on the extraction of Cd^2+^ and Ni^2+^. The logD value increases linearly with an increasing initial concentration of ammonia with a slope of 2. These findings suggest that a 2:1 (H_8_L: metal) complex was extracted into the organic phase by releasing an equimolar amount of protons from H_8_L along with two ammonia molecules.

**Figure 6:**
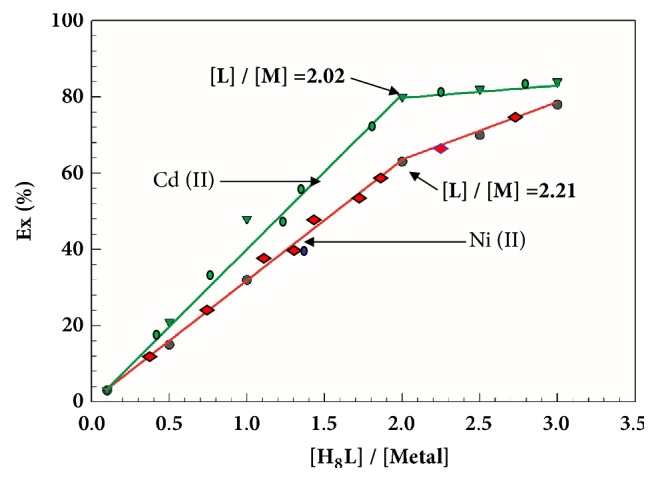
Plots of percentage extraction vs. molar ratio [L]/[M] for the extraction of Cd^2+^ and Ni^3+^ with H_8_L under the same reaction condition of Figure 3. O:A = 1:1. Symbol (empty green inverted triangle), (filled green circle) (repeated) (filled red diamond) (filled red circle) (repeated)-H_8_L; O/A = 1; T = 25°C.

Distribution experiments were carried out in order to obtain information on the viability of the extraction process, the stoichiometry and distribution equilibrium of the extracted metal ions between phases, and the extent of Cd^2+^ and Ni^2+^ extraction. During this experiment, the concentrations of Cd^2+^ and Ni^2+^ were kept constant at 1 × 10^−3^ M, while the concentration of the extractant was varied from 1 × 10^−4^ to 2 × 10^−3^ M. This means that the relative concentration of the extractant H_8_L (defined as the molar ratio of the initial extractant concentration and the concentration of the extracted metal) changed from 0.1 to 3.4 (Figure 6). A plot of the residual concentration of Cd^2+^ and Ni^2+^ in the aqueous phase, against the relative concentration of extractant, is presented in Figure 6. Initially, experiments were completed three times to examine their reproducibility. These results are also presented in Figure 6. As can be seen, the extraction method showed good reproducibility. An extraction percentage higher than 75% was obtained using H_8_L at low concentrations of Cd^2+^ and Ni^2+^. For Cd^2+^ and Ni^2+^ extracted by the same reaction condition, the higher Ex % of Cd^2+^ must have a high extraction equilibrium constant. That is why the slope of straight line after the inflection point in Figure 6 becomes more horizontal in green line (Cd^2+^) than red line (Ni^2+^).

As mentioned above the molar ratio of H_8_L: metal was 2:1. These extraction studies suggest that the 2:1 (H_8_L: metal) complex was extracted into the organic phase releasing 1 M protons from 1 M H_8_L, accompanying two ammonia molecules [Figures 4 and 5]. The extraction equilibrium and the extraction equilibrium constant K_ex_ can be expressed as

(2) MNH3i2++2H8Lo+2NH3=MNH3i+2H6Lo+2H+

(3) Kex=MNH3i+2H6LoH+2+MNH3i2+H8Lo2NH32

where (o) indicates the species in the organic phase. Equation (3) can be simplified as follows using the distribution ratio of the metal (D = [M(NH_3_)_i+2_H_8_L]_o_ / [M(NH_3_)_i_^2+^]):

(4) Kex′=DH2H6Lo2NH32

(5) log⁡D=log⁡Kex′+2log⁡H8Lo+2pH+2logNH3

From the above observations, it is suggested that the ammonia molecules and amine complex participate in the extraction of transition metal ions with H_8_L.

The extraction of transition metal ions with H_8_L from the aqueous phase, containing 0.1 M ethylenediamine [C_2_H_4_(NH_2_)_2_] and 0.1 M trimethylenediamine [(CH_2_)_3_(NH_2_)_2_] instead of ammonia, into the dichloromethane solution was also examined. It is worth noting that extraction of Co^2+^ and Ni^2+^ from the aqueous phase containing ethylene diamine was suppressed (Ex% = 0). On the other hand, Cu^2+^ and Cd^2+^ were extracted remarkably well (Cu^2+^ = 100% and Cd^2+^ = 90%) from the aqueous phase containing ethylene diamine. The interaction of calixarenes and amines in the dichloromethane solution likely involves the following two-step process: (i) proton transfers from the calixarene to the amine to form the amine cation and then (ii) the calixarene anion forms an endo-calix complex by association with the amine cation [35]. However, we think these reactions simultaneously take place and reach equilibrium at certain conditions.

To understand complexation in the aqueous phase, the distribution ratio of the M(en)_2_ and M(en)_3_ [(en)_2_= ethylene diamine and (en)_3_ = trimethylene diamine] species in the aqueous phase about each metal ion before extraction, using the formation constant with ethylene di and triamine, was calculated.  Table 1 shows the distribution ratios of the M(en)_2_ and M(en)_3_ species in the aqueous phase before extraction and the extraction percentage for extraction with H_8_L into dichloromethane. The distribution ratios for the complexations with H_8_L are comparable and indicative of high efficiency (Table 1). The values of the distribution ratios of M(en)_2_ and M(en)_3_ species in the aqueous phase before extraction are integers. As mentioned above, the extraction of Co^2+^ and Ni^2+^ metal ions was suppressed. Thus, most of the existing species in the aqueous phase are M(en)_3_, while few M(en)_2_ species were present, whereas in the case of Cu^2+^ most of the extracted species present in the aqueous phase were M(en)_2_ species. These data suggest that the existence of M(en)_2_ species diverts and/or controls the extraction with H_8_L from the aqueous phase containing ethylene diamine to some extent. This can be explained by steric factors influencing the binding of the ligands to the metal ions. It means that probably the small molecule are encapsulated by larger molecular and steric barriers keep the guest from escaping the host. From the above results, masking effects of metal ions with amines were also observed, particularly metal ions showing high affinity with amines.

We additionally studied the composition of the Cu^2+^ and Cd^2+^ extracted species according to the molar ratio method. A plot of logD vs. log[H_8_L] was constructed and a straight line was found with a slope of 1, indicating a molar ratio of H_8_L:metal = 1:1, which is different from the composition of species in the case of the ammonia aqueous phase. The effect of pH on the extraction of Cu^2+^ was also verified. As logD increases linearly with an increase in pH, where a slope of 2 was obtained.

Thus, the extraction equilibrium can be expressed as (en= H_2_N(CH_2_)_2_NH_2_)

(6) $$\begin{array}{c} {M_{\left(en\right)2}}^{2+}+H_{8}L_{\left(O\right)}=M_{\left(en\right)2}H_{6}L_{\left(O\right)}+2H^{+} \end{array}$$

Table 2 shows the elemental analysis data of the Cu^2+^-H_8_L and Cd^2+^-H_8_L complexes. These data indicate that the extracted species from the aqueous phase containing ethylene diamine was M(en)_2_ not M(en)_3_, in agreement with our initial assumption based on Table 1.

**Scheme 1:**
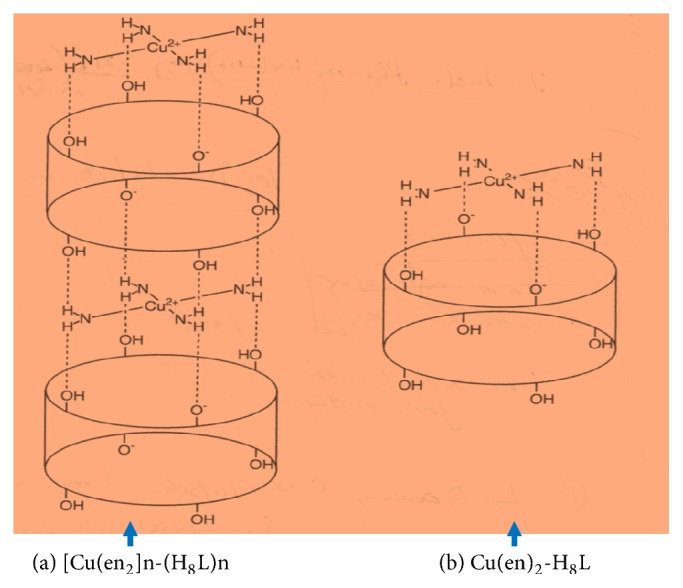
Structure of Cu(en)_2_-H_8_L complex.

It has been reported that, in the extraction of metal ions using calix[n]arenes, the metal ion selectivity is related to the ring size of the calix[n]arene and to the radii of the metal ions [9, 10]. The present extraction study using H_8_L accompanied by amines indicated that size is key to the selectivity, as the diameter of the H_8_L ring (4-4.4 Å) is sufficient to fit the M(en)_2_ complex (where the distance between the both ends of amino proton is ca. 4.0 Å). A possible structure of Cu^2+^-H_8_L is given in Scheme 1. An X-ray results is essential to determine a compound structure. At present, our research is now progressing in this direction.

**Figure 7:**
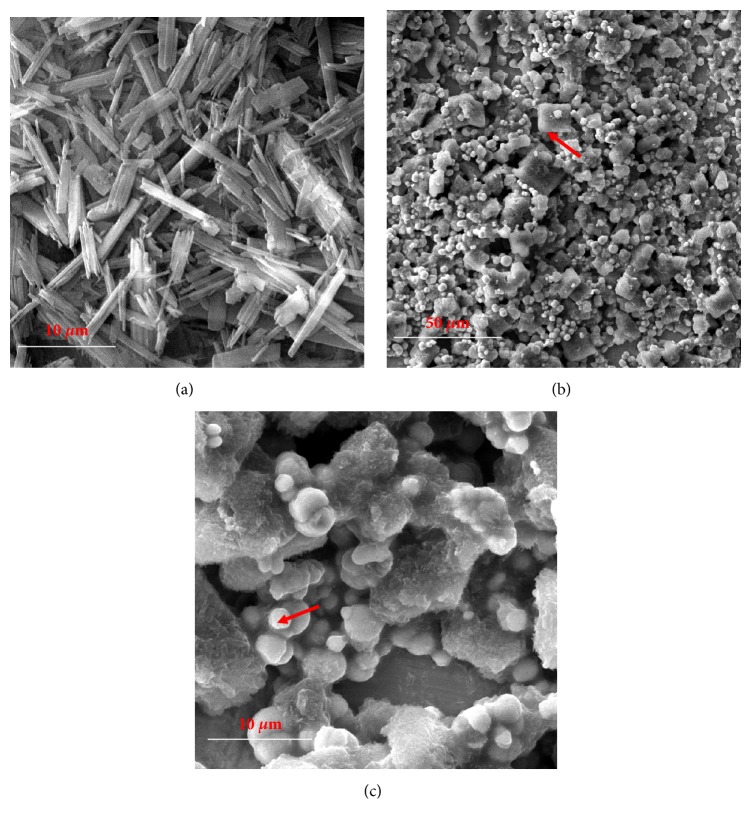
FESEM images of the samples: (a) H_8_L, (b) Cd^2+^-H_8_L, and (c) sample (b) at high magnification.

**Figure 8:**
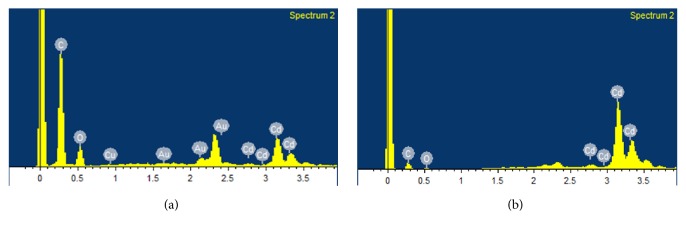
EDX spectra of (a) H_8_L and (b) Cd^2+^-H_8_L. EDX spectra for the region marked by an arrow in (b), and (c) of Cd^2+^-H_8_L in Figure 7.

As mentioned above Cd^2+^ is very toxic and inhaling cadmium dust leads to kidney problems which can be fatal. Therefore, complexation studies of Cd^2+^-H_8_L complexes were also performed using FESEM, FTIR, and ^1^H NMR spectroscopy for more information of Cd^2+^ extraction with H_8_L. FESEM images of H_8_L and Cd^2+^-H8L showed very unique surface morphology. The H_8_L had rod-like particles with almost 10 *μ*m long and less than 1 *μ*m wide [Figure 7(a)]. However, the Cd^2+^-H_8_L morphology was totally changed in compare with H_8_L alone. The Cd^2+^-H_8_L sample had two types of particle sizes as shown in Figures 7(a) and 7(b), respectively. The nanosize spherical particles were Cd^2+^ ions and the square or bigger size particles were H_8_L as evidence by EDX analysis. The EDX and elemental analysis showed almost the same results. It is interesting that the Cd^2+^ ions were homogeneously dispersed and/or distributed over the H_8_L ligand.  Figure 8(a) shows the EDX spectrum of big particles marked by red arrow in Figure 7(b). The atomic percentage of C was 69.26 for the big size particles. Very little amount of Cd^2+^ was also obtained during EDX analysis of big size particles. On the other hand, the spherical particles had mostly Cd^2+^ ion and atomic percentage of Cd was 33.78 [Figure 8(b), the EDX spectrum of spherical particle was based on Figure 7(c), marked by red arrow]. Almost the same results were obtained for different location based on particles size. The EDX results shown in Figures 8(a) and 8(b) indicate the presence of Cd, C, Au, Cu, and O. The Au and Cu were found due to gold coating over the sample and Cu substrate was used.

**Figure 9:**
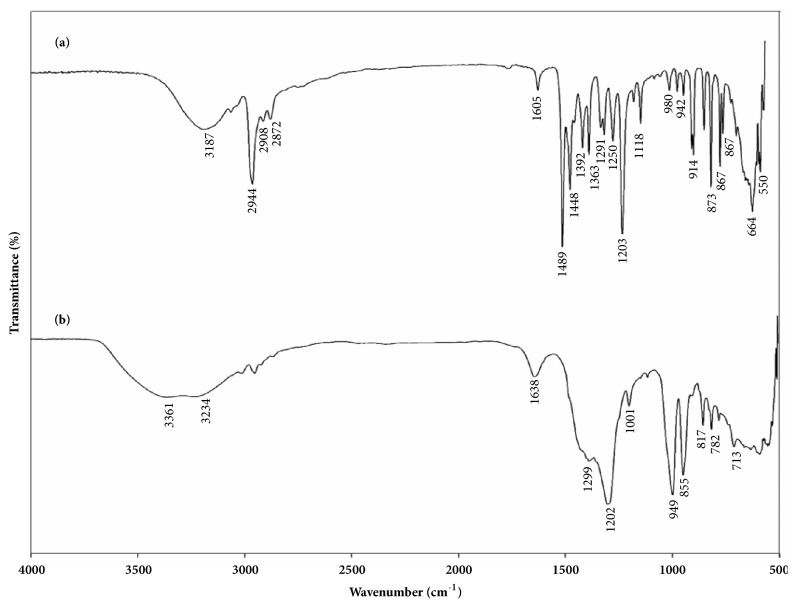
FTIR spectra of (a) H_8_L and (b) Cd^2+^-H_8_L.

The FTIR spectrum of H_8_L and Cd^2+^-H_8_L is shown in Figures 9(a) and 9(b), respectively. In the FTIR spectra of the complexes, the intensities or wave numbers of the stretching vibration of the OH groups change drastically with complexation by breakage of the especially strong intramolecular hydrogen bonding existing in the free ligands [30]. The IR absorption bands at 3187 cm^−1^ due to *υ*N-H: free ethylene diamine [36] shifted to 3361 cm^−1^ upon extraction of Cd^2+^ with H_8_L. This indicates that hydrogen bonding was likely present in the M(en)_2_-H_8_L complex. The associated natures of C(CH_3_)_3_, –CH_2_–, and hydroxyl group have been reported previously [30]. The band C(CH_3_)_3_ gives a sharp absorption band at ca. 2944 cm^−1^, and this peak intensity was weakened after making a complex with Cd^2+^ cation. The *ν*(C=C) vibration bands shift by about 30 cm^−1^ (i.e., from 1605 to 1638 cm^−1^) toward higher frequencies. It has been also recorded that the –CH_2_– vibrations almost disappeared and the very strong peak at 1489 cm^−1^ was disappeared. The differences in the infrared spectra may be caused by hydrogen bonding. As a whole, all the peaks of Cd^2+^-H_8_L composites were shifted to higher energy direction, indicating the strong interaction between Cd^2+^ and H_8_L.

**Figure 10:**
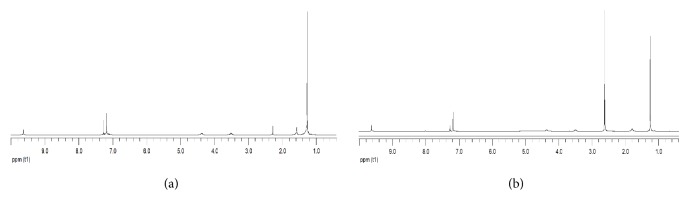
^1^H NMR spectra of (a) H_8_L at 25°C in CDCl_3_ and (b) Cd-H8L at 25°C in CDCl_3_ at 400 MHz.

The solution behavior of the complexes was determined by ^1^H-NMR spectroscopy in CDCl_3_ at room temperature. In the spectra of H_8_L [Figure 10(a)], and Cd^2+^-H_8_L [Figure 10(b)], each spectrum shows one singlet resonance for protons of -C(CH_3_)_3_ at *δ* = 1.25. For H_8_L, singlet resonances of Ar-H are observed at *δ* = 7.12 and 7.14 ppm, respectively; for Ar-OH these resonances are at *δ* = 9.63, respectively. A new signal appeared at chemical shift of *δ* = 2.62 which was not observed in the H_8_L. In our previous study, we also observed one singlet at around *δ* 3.00 for Ce^3+^-H_8_L and Ce^3+^-H_6_L probably for the bridging methane groups due to the different environments of the hydrogen atoms on the methylene group [30]. In the ^1^H NMR spectra, peaks at 3.5 and 4.4 ppm were observed (due to the nonequivalent methylene protons (Ar-CH_A_H_B_-Ar) in the free H_8_L which converge to 3.8 ppm in the Cd(en)_2_-H_8_L complex after extraction. This indicates that the cone conformation of H_8_L converts into the 1,3,5,7-alternate conformation.

**Figure 11:**
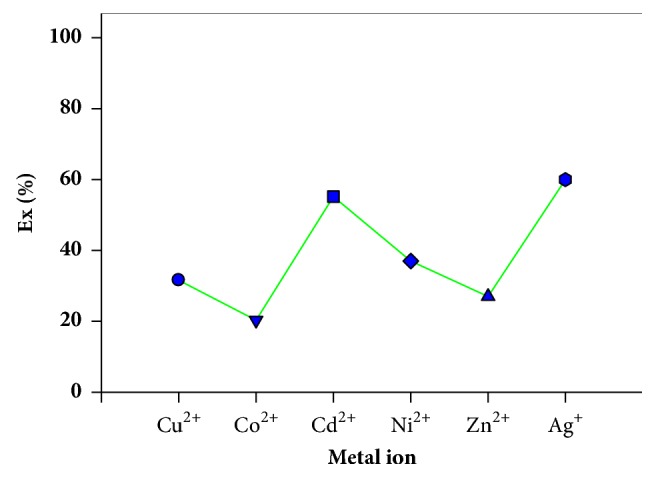
Metal ions percentage extraction (E%) at pH 11.5 by H_8_L-ester. O/A = 1; T = 25°C.

In this study, the H_8_L-ester compounds were also synthesized according to published procedures [37]. The extraction percentage of any metal ion was poor throughout the entire range of pH used. In fact, the liquid–liquid extraction ability of transition metal ions with the H_8_L-ester/CH_2_COOC_2_H_5_ derivative was low in comparison with H_8_L alone. It is thought that the H_8_L becomes an anion due to deprotonation. The H_8_L-ester, which is modified from calix[8]arene, is more flexible than both the calix[4]arene and calix[6]arene derivatives; however, it did not show a good extraction capability for Co^2+^, Ni^2+^, Zn^2+^, and Ag^+^, as shown in Figure 11.

## 4. Conclusions

The host–guest extraction and/or complexation of divalent heavy metal cations and silver by H_8_L and its ethyl ester host was investigated by changing various experimental parameters. The effect of the organic solvent on the extraction procedure was examined. In the solvents, the affinity of H_8_L to bind transition metal cations was found to be much higher in the case of Cd^2+^ and Ni^2+^ when ammonia was used as the aqueous phase. The results from the extraction study suggested that, in the case of ammonia, the ratio of the extracted species is 2:1 (H_8_L:metal), whereas in the presence of ethylene diamine instead of ammonia, the composition of the extracted species is 1:1, indicating selectivity towards tetrahedral type metal ions. The ester-H_8_L compounds were found to be not effective for the extraction of transition metal cations under the present reaction conditions. The extraction behavior of the metal ions was closely related to the pH at equilibrium and the matrix and/or medium (i.e., ammonia or amine) of the aqueous solution and organic phase.

## Figures and Tables

**Table 1:** Extraction percentage (Ex%) of the transition metal ions with H_8_L from ethylene diamine into dichloromethane at 25°C and distribution ratio of M(en)_2_ and M(en)_3_ in the aqueous phase before extraction. Uncertainties are given in parentheses as standard errors of the mean (N = 3).

	%E	M(en)_2_^*∗*^	M(en)_3_^*∗∗*^
		(%)	(%)
Co(II)	0	0	100
Ni(II)	0	0	100
Cu(II)	97.0 (2)	100	40
Zn(II)	46.0 (3)	3	97
Ag(I)	52.7 (5)	99	35
Cd(II)	90.1 (1)	15	85

*∗* **(en)**_**2**_ **= **ethylene diamine,*∗∗ ***(en)**_**3**_** = **ethylene triamine.

**Table 2:** Elemental analysis of Cu^2+^-H_8_L and Cd^2+^-H_8_L complexes and estimated chemical formula.

Cu^2+^: estimate chemical formula				Cd^2+^: estimate chemical formula			
Cu(en)_2_H_6_L.5H_2_O				Cd(en)_2_ H_6_L.3H_2_O			

H(%) C(%) N(%)				H(%) C(%) N(%)			

Obs.	8.37	70.89	3.56	Obs.	8.18	69.90	3.49

Calc.	8.73	70.40	3.57	Calc.	8.41	69.83	3.54

## Data Availability

The data used to support the findings of this study are available from the corresponding author upon request.
